# Dissecting the cytochrome *c*_2_–reaction centre interaction in bacterial photosynthesis using single molecule force spectroscopy

**DOI:** 10.1042/BCJ20170519

**Published:** 2019-08-09

**Authors:** Cvetelin Vasilev, Guy E. Mayneord, Amanda A. Brindley, Matthew P. Johnson, C. Neil Hunter

**Affiliations:** Department of Molecular Biology and Biotechnology, University of Sheffield, Sheffield S10 2TN, U.K.

**Keywords:** atomic force microscopy, cytochrome, electron transfer, photosynthesis, reaction centre

## Abstract

The reversible docking of small, diffusible redox proteins onto a membrane protein complex is a common feature of bacterial, mitochondrial and photosynthetic electron transfer (ET) chains. Spectroscopic studies of ensembles of such redox partners have been used to determine ET rates and dissociation constants. Here, we report a single-molecule analysis of the forces that stabilise transient ET complexes. We examined the interaction of two components of bacterial photosynthesis, cytochrome *c*_2_ and the reaction centre (RC) complex, using dynamic force spectroscopy and PeakForce quantitative nanomechanical imaging. RC–LH1–PufX complexes, attached to silicon nitride AFM probes and maintained in a photo-oxidised state, were lowered onto a silicon oxide substrate bearing dispersed, immobilised and reduced cytochrome *c*_2_ molecules. Microscale patterns of cytochrome *c*_2_ and the cyan fluorescent protein were used to validate the specificity of recognition between tip-attached RCs and surface-tethered cytochrome *c*_2_. Following the transient association of photo-oxidised RC and reduced cytochrome *c*_2_ molecules, retraction of the RC-functionalised probe met with resistance, and forces between 112 and 887 pN were required to disrupt the post-ET RC–*c*_2_ complex, depending on the retraction velocities used. If tip-attached RCs were reduced instead, the probability of interaction with reduced cytochrome *c*_2_ molecules decreased 5-fold. Thus, the redox states of the cytochrome *c*_2_ haem cofactor and RC ‘special pair’ bacteriochlorophyll dimer are important for establishing a productive ET complex. The millisecond persistence of the post-ET cytochrome *c*_2_[oxidised]–RC[reduced] ‘product’ state is compatible with rates of cyclic photosynthetic ET, at physiologically relevant light intensities.

## Introduction

Biological membranes house a wide variety of proteins involved in electron transfers (ETs), ion and peptide transport, signalling and enzyme reactions. One crucial aspect of membrane protein function is the interaction with an extrinsic partner, for example, as part of a linked series of catalytic reactions, the transfer of electrons, or the binding of a ligand to a membrane receptor. In bioenergetic membranes extrinsic partners are generally small, diffusible redox proteins such as *c*-type cytochromes in mitochondria and phototrophic bacteria, and plastocyanin in cyanobacteria and plants. Such proteins facilitate ETs in respiration and photosynthesis by docking onto protein complexes at the membrane interface in order to donate or receive electrons. Bacterial, mitochondrial and photosynthetic ET chains are housed within membranes, the infoldings of which confine these small redox proteins within a lumen, assisting their rapid diffusion at or near the membrane surface and promoting their ability to shuttle electrons between membrane-bound complexes on a millisecond timescale.

The spherical intracytoplasmic membranes (‘chromatophores’) found in the phototrophic bacterium *Rhodobacter* (*Rba.*) *sphaeroides* provide an ideal experimental system for investigating the docking/undocking events involved in ET between membrane intrinsic and extrinsic redox partners. This bacterium synthesises hundreds of invaginated vesicular chromatophores, which can detach from their cytoplasmic membrane anchoring points and exist as vesicles of ∼50–60-nm diameter [1–3]. Purified chromatophore vesicles have been analysed using electron microscopy and atomic force microscopy (AFM) [4,5], and the constituent major membrane protein complexes have been characterised extensively by structural and kinetic methods [6–10]. This large body of work has culminated in a structural and functional model for a whole membrane vesicle, comprising 63–67 light-harvesting 2 (LH2) complexes, 11 reaction centre light-harvesting 1-PufX (RC–LH1–PufX) dimers, 2 RC–LH1–PufX monomers, 4 cytochrome *bc*_1_ (cyt*bc*_1_) dimers and 2 ATP synthases [4,11]. The 100-million atom model [11] accounts for an interlinked series of processes, from the absorption of light and excitation energy transfer by antenna complexes, to charge separation at the RC, then quinone diffusion to the cyt*bc*_1_ complex and the generation of a protonmotive force, and finally to consumption of the protonmotive force by the ATP synthase, generating ATP.

**Figure 1.**
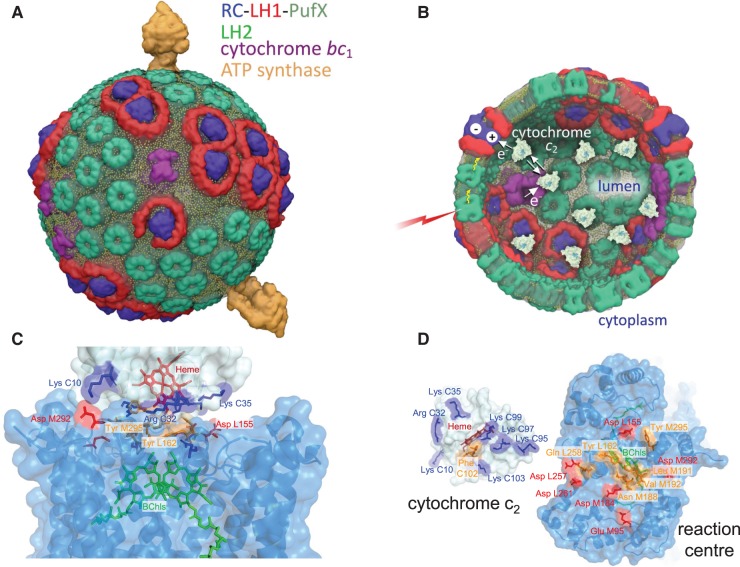
Molecular models of the chromatophore membrane vesicle that houses cytochrome *c*_2_ and of the cytochrome binding site on the RC. (**A**) A chromatophore vesicle, comprising 63 LH2 antenna complexes (green), 11 dimeric and 2 monomeric RC–LH1–PufX complexes with LH1 (red) and RC (blue). There are also 4 cyt*bc*_1_ dimers (purple) and 2 ATP synthase complexes (orange). These photosynthetic complexes are embedded in a membrane of ∼17 000 lipid molecules (lipid phosphates are indicated in yellow). This figure was produced by Dr Abhishek Singharoy. (**B**) View of the chromatophore interior, depicting 9 cytochrome *c*_2_ molecules (pale blue) out of a total of ∼12 that reside in the lumen [12]. This diagram also depicts excitation of an LH2 complex by light (red), excitation transfer to LH1 (yellow wavy arrows), charge separation at a RC and the role of cytochrome *c*_2_ as a carrier of electrons from the cyt*bc*_1_ complex to the RC. This cyclic electron transfer pathway results in protons transiently accumulating within the chromatophore lumen; the ATP synthases consume the protonmotive force and form the last stage in the overall process of converting the energy of light into ATP. (**C**) The binding interface between the RC and cytochrome *c*_2_, as revealed by the cytochrome *c*_2_:RC co-crystal structure (PDB 1L9B). The cytochrome surface is shown in pale blue, and the RC surface in blue. Acidic residues are highlighted in red, basic residues are in dark blue and hydrophobic regions are in orange. The cytochrome haem is shown in dark red and the photo-oxidisable bacteriochlorophyll dimer, BChl_2_, is shown in green. (**D**) The RC-facing surface of cytochrome *c*_2_ and the periplasmic (lumenal) face of the RC complex, with colours as shown in (**C**).

The chromatophore (Figure 1A) houses a simple, cyclic ET pathway, driven by the energy of photons harvested by antenna complexes. This energy migrates to the sites of photochemistry, the RC complexes, where a charge separation is followed by a series of electron then proton transfers that culminate in a doubly reduced quinone acceptor, QH_2_. The lumen of the chromatophore vesicle contains cytochrome *c*_2_ molecules (Figure 1B), and docking of reduced cytochrome *c*_2_ onto the periplasmic surface of the photo-oxidised RC triggers ET, which resets the RC for another charge separation. Oxidised cytochrome *c*_2_ undocks from the RC and diffuses to the nearby cyt*bc*_1_ complex [12], the turnover of which limits the rate of ATP synthesis [11]. Repeated turnovers of this interlinked cyclic bioenergetic system are therefore sustained by a constant input of light, the rapid shuttling of cytochrome *c*_2_ and quinone back and forth between RC and cyt*bc*_1_ complexes, and turnover of the ATP synthase complex, which stores solar energy in a chemical form as ATP. The chromatophore is configured for low-light illumination, with 79% of the maximal rate of ATP production possible at only 1% of the maximum possible light intensity [11,13]. Figure 1A,B summarises the morphology, composition and function of an intracytoplasmic membrane from (*Rba.*) *sphaeroides*, adapted from the study by [4,11].

This study focuses on one aspect of this bioenergetic system, the binding/unbinding of cytochrome *c*_2_ onto the RC. The crystallographic structure of the cytochrome *c*_2_–RC complex revealed molecular details of the interface between these proteins [14,15], which contains a central domain with limited solvent accessibility comprising hydrophobic, hydrogen bonding and cation–π interactions. This region is surrounded by a more solvent-accessible electrostatic domain in which positively charged residues on cytochrome *c*_2_ sit opposite negatively charged residues on the periplasmic surface of the RC (Figure 1C,D). Solvation of the charged side chains leads to a ∼4.5 Å separation between these charged residues [14,15]. This static view of the cytochrome *c*_2_–RC complex has been augmented by rapid kinetic measurements of ET between these proteins (reviewed in [16]), determined at a range of ionic strengths using purified components, photosynthetic membranes, and a variety of site-directed mutants that probe the cytochrome *c*_2_–RC interface [17–23]. Following a single flash that photo-oxidises the RC, there is a fast, ∼1 μs, transfer of an electron from cytochrome *c*_2_, ascribed to a state where the cytochrome is ‘pre-bound’ to its site on the RC before the flash. A slower 50–200-μs phase in such kinetic assays likely reflects a series of processes that include the electrostatically guided approach of the cytochrome arriving from a bulk phase forming an initial encounter complex [24], and its orientation to present the haem edge towards the periplasmic surface of the RC. The final docked configuration of the ET complex is assumed to resemble that found for the structure of the cytochrome *c*_2_–RC complex, in which the proximal edge of the haem of the electron donor, cytochrome *c*_2_, is 8.4 Å from the uppermost edge of the BChl dimer of the RC, the electron acceptor. However, the conjugated atoms in these cofactors are separated by 14.2 Å; the aromatic ring of the RC residue, Tyr L162, intervenes between the haem and BChls, and it has been proposed that this residue creates a tunnelling pathway for ET between these proteins [15,25,26].

The formation of a transiently stable complex is essential for rapid ET, and the structure of the cytochrome *c*_2_–RC complex shows that Tyr L162, Leu M191 and Val M192 on the RC and Phe C102 and the haem methyl group on cytochrome *c*_2_ are in van der Waals (VDW) contact [14–16]. However, equal consideration should be given to the post-ET events that involve the unbinding of oxidised cytochrome *c*_2_ (cytochrome *c*_2_[ox]) from the reduced RC (RC[red]), since it is functionally essential that cytochrome *c*_2_[ox] returns to the cyt*bc*_1_ complex to pick up an electron. Gerencsér et al. [27] mixed horse heart cytochrome *c* and detergent-solubilised RC-only complexes (with no surrounding LH1 complex) in solution and showed that dissociation of the ET complex is the bottleneck of cytochrome turnover. Several other studies showed preferential binding of cytochrome *c*_2_[ox] or cytochrome *c*[ox] to RCs [28], to the extent that the oxidised cytochrome impeded the access of cytochrome *c*_2_[red] to its docking site on the RC [19,20].

Steered molecular dynamics (SMD) simulations of the RC–cytochrome *c*_2_ complex specifically address the issues of docking [29], and the exit strategy for cytochrome *c*_2_ as it dissociates from the RC [30]. The post-ET RC[red]–cytochrome *c*_2_[ox] complex has also been studied at the single-molecule level using AFM; PeakForce quantitative nanomechanical imaging (PF-QNM) was used to record interactions between cytochrome *c*_2_ molecules tethered to an AFM probe and single RC–LH1–PufX core complexes immobilised onto a functionalised gold substrate [31]. A force of 480 pN was required to pull apart the RC[red]–cytochrome *c*_2_[ox] complex, a surprising result given that in the immediate aftermath of ET, cytochrome *c*_2_[ox] might be expected to dissociate spontaneously from the RC. Here, we use two force spectroscopy approaches to investigate the intermolecular forces that govern the cytochrome *c*_2_–RC ET complex, at the single-molecule level and under physiological conditions. We validate the specificity of recognition between tip-attached RCs and surface-tethered cytochrome *c*_2_ using microscale patterns of cytochrome *c*_2_ and the cyan fluorescent protein (CFP), and use force spectroscopy, with varying loading rates, to calculate *k*_off_ values for dissociation of the cytochrome *c*_2_–RC complex. Our results are discussed in relation to the kinetics of the cyclic ET pathway found in phototrophic bacteria.

## Materials and methods

### Protein purification

*His-tagged CFP* — The gene sequence of CFP was amplified by PCR from SCFP3A vector (Addgene plasmid 22905) [32]. The resulting *Nde*I/*Bam*HI fragment was cloned into a pET14b expression vector (Novagen). The His_6_-CFP protein was produced by heterologous expression in *Escherichia coli* (BL21); cells were grown to an OD_680_ of 0.6 at 37°C and then induced using IPTG (0.4 mM) for 12 h at 25°C. Pelleted cells (19 000g/20 min) were lysed by sonication, and the resulting lysate was clarified by a further spin (33 000g/30 min). The His-tagged fluorescent protein was purified to homogeneity from clarified lysate using a Chelating Sepharose Fast Flow Ni-NTA gravity flow column (GE Healthcare) as detailed in the manufacturer's instructions. Protein purity was assessed by gel electrophoresis (SDS–PAGE).

*The RC–His*_12_*–LH1–PufX complex* — A *Rba. sphaeroides* Δ*puhA* mutant capable of synthesising a RC with its H-subunit bearing a His_12_-tag at the carboxyl terminus [31] was a kind gift from Professor Tom Beatty (University of British Columbia). This strain was grown semi-aerobically in 1.5 l of M22 liquid culture containing 1 mg ml^−1^ of tetracycline at 34°C for 2 days in a shaker incubator (in the dark at 180 rpm). The 1.5-l culture was harvested by centrifugation (5300***g***/25 min in a Beckman JA-10 rotor at 4°C), and the cell pellet was resuspended in 15 ml of 10 mM HEPES of pH 7.4 buffer. The concentrated cell suspension was frozen at −20°C until immediately prior to French press disruption. The cells were pretreated with lysozyme (0.7 mg/ml final concentration) and incubated at 37°C for 30 min. DNase I was added to the cells prior to lysis and the pressing was conducted at a cell pressure of 2.9 MPa in an Aminco French pressure cell. The pressing was repeated for maximum lysis. The lysate was loaded onto a 15%/40% (w/w) sucrose step gradient and centrifuged in a Beckman Ti 45 rotor for 10 h at 57 000***g*** at 4°C. The intracytoplasmic membrane fraction was harvested from the interface and further treated to concentrate the membranes by diluting out the sucrose with 10 mM HEPES of pH 7.4 buffer and centrifuging in a Beckman Ti 45 rotor for 2 h at 125 000***g*** at 4°C. The membrane pellet was resuspended in a small volume, typically 1 ml of 10 mM HEPES of pH 7.4 buffer, and frozen at −20°C for further use. The membrane pellet obtained from sucrose gradient centrifugation were solubilised with *n*-Dodecyl-*beta*-d-maltoside (β-DDM, Glycon) at a final concentration of 59 mM, and a final OD of the membrane sample of ∼60 at 875 nm. The mixture was stirred at 4°C in the dark for 90 min. Non-solubilised material was removed by centrifugation (in a Beckman Ti 45 rotor for 2 h at 125 000***g***), and the supernatant was loaded onto Chelating Sepharose Fast Flow Ni-NTA column (GE Healthcare) equilibrated with 10 mM HEPES of pH 7.4, 500 mM NaCl, 10 mM Imidazole and 0.59 mM β-DDM buffer. A gradient of 10–400 mM Imidazole was applied and the main peak, which contains pure His_12_–RC–LH1–PufX, appeared when the concentration of Imidazole reached ∼300 mM. Eluted protein was concentrated (Vivaspin 500 spin-concentrator, Sartorius) and dialysed against 10 mM HEPES of pH 7.4, 50 mM NaCl and 0.59 mM β-DDM buffer. Then, the RC–LH1–PufX protein (henceforth RC–LH1–PufX) was loaded onto a DEAE-Sepharose (Sigma) ion-exchange column equilibrated with 10 mM HEPES of pH 7.4, 50 mM NaCl and 0.59 mM β-DDM buffer. A gradient of 50–300 mM NaCl was applied with the main peak of pure protein appearing at NaCl concentration of ∼280 mM. The best fractions judged from the peak absorbance ratio of 875–280 nm were pooled (*A*_880_/*A*_280_, ∼1.9). The protein was again concentrated and dialysed against 10 mM HEPES of pH 7.4, 50 mM NaCl, and 0.59 mM β-DDM buffer and applied to a HPLC column (Phenomenex BioSep) and eluted at a flow rate of 0.3 ml min^−1^ in order to separate the monomeric and dimeric RC–LH1–PufX complexes. The second elution peak (corresponding to monomeric fraction of RC–LH1–PufX) was collected, concentrated to a final concentration of 15 µM in 10 mM HEPES of pH 7.4, 50 mM NaCl, 0.59 mM β-DDM buffer and stored at −80°C for further use.

*Cyt c*_2_–*His*_6_ — The cytochrome *c*_2_ gene was cloned from *Rba. sphaeroides* strain 2.4.1 using a 5′ primer TCGAATTCATGTCATGCATGATCCGGAACG, which contains an *Eco*RI site for cloning into the pRK415 plasmid and a 3′ primer AAGCTTTCAGTGGTGGTGGTGGTGGGGGGCCGGACGGCGACCTGCTGG which includes a His_6_ sequence for immobilised metal ion affinity chromatography (*IMAC*) and a *Hin*dIII site for cloning into pRK415 plasmid. The gene was cloned using Touchdown PCR and sub-cloned into the pRK415 vector using *Eco*RI and *Hin*dIII restriction sites for directional cloning. The plasmid with the gene was then mated into a Δ*cycA* strain of *Rba. sphaeroides* via *E. coli* S17 [33]. The intracytoplasmic membrane fraction from the cytochrome *c*_2_*-*His_6_ mutant was prepared as described in the previous paragraph. The membrane pellet obtained from sucrose gradient centrifugation was solubilised with *N*,*N*-dimethyldodecylamine *N*-oxide (LDAO, Fluka) at a final concentration of 65 mM, and a final OD of the membrane sample of ∼80 at 875 nm. The mixture was stirred at room temperature in the dark for 20 min. Non-solubilised material was removed by centrifugation (in a Beckman Ti 45 rotor for 2 h at 125 000***g***), and the supernatant was loaded onto Chelating Sepharose Fast Flow Ni-NTA column (GE Healthcare) equilibrated with 10 mM HEPES of pH 7.4, 500 mM NaCl, 10 mM Imidazole, 1 mM LDAO buffer. A gradient of 10–400 mM imidazole was applied and the purified cytochrome *c*_2_-His_6_ eluted when the concentration of imidazole reached ∼270 mM. The purified protein (*A*_414_/*A*_280_ ratio of ≥3.3) was dialysed against 10 mM HEPES of pH 7.4, 50 mM NaCl, 1 mM LDAO buffer, concentrated to a final concentration of 740 µM, and stored at −80°C for further use.

### Functionalisation of AFM probes and sample substrates

Si wafers ([100] polished face, 625-µm thickness from Prolog Semicor Ltd) were cleaved into small substrates (SiO*_x_* substrates), typically 5 × 5 mm^2^. Then, the substrates were cleaned in Piranha solution [H_2_O_2_/H_2_SO_4_ 1:2 (v/v)] for 1 h, rinsed 10 times in deionised (DI) water (NANOPure Diamond, Barnstead) and finally dried in a pure nitrogen stream. Hybrid AFM probes, Si tip mounted on a Si_3_N_4_ rectangular or triangular cantilever, model BL-AC40TS-C2 (Olympus Probes) or MSNL (Bruker), respectively, were first cleaned by washing in acetone (HPLC grade from Fisher Scientific) and then cleaned using an in-house-built UV/Ozone cleaner (LSP035 Pen-Raylight source, LOT-Oriel Ltd) for 45 min. Immediately after the cleaning step, both the Si substrates and the AFM probes were placed into a glass desiccator and coated with self-assembled monolayer (SAM) of 3-mercaptopropyltrimethoxysilane (MPTMS, Sigma–Aldrich) using a simple vapour-phase method: Both the AFM probes and the SiO*_x_* substrates were placed into a glass desiccator, purged with pure nitrogen for 10 min and then 20 µl of MPTMS was introduced into the desiccator. After another 5-min purge, the desiccator was evacuated down to ∼300 Pa using a dry mechanical pump (Welch, model 2027) and then sealed for 6–8 h to facilitate the deposition of the SAM. The quality of the SAM formed on the SiO*_x_* substrate was determined by static contact angle and AFM measurements. The water contact angle measured was 63 ± 7° which is consistent with the reported values for closely packed MPTM SMAs [34]. AFM measurement also revealed a surface roughness of ∼0.11 nm, a value nearly identical with that measured for the native oxide surface of a Si wafer, thus indicating a comprehensive coverage of the monolayer film. We assumed the same quality of the monolayer formed on the tips of the AFM probes since they have surface properties identical to the Si wafers.

The next step in the functionalisation of the AFM probes, immediately after the SAM formation, was to introduce the cross-linker used to introduce Ni^2+^-NTA functionality. This, in turn, was used to bind His_12_–RC–LH1–PufX complexes to the AFM tip. We used an amine-to-sulfhydryl heterobifunctional cross-linker with a 5.5-nm-long polyethylene-glycol (PEG) spacer arm, an *N*-hydroxysuccinimide (NHS) ester group at one end and a maleimide (MAL) group at the other end (SM(PEG)_12_, Pierce Biotechnology) in order to attach *N*-(5-amino-1-carboxypentyl)iminodiacetic acid (AB-NTA, Dojindo Laboratories) molecules to the mercapto-functionalised AFM probe. First, a 50 mM stock solution of AB-NTA in DI water was prepared. The NTA functional group was charged with Ni^2+^ ions by adding NiSO_4_ in the stock solution to a final concentration of 70 mM. Also, a 250 mM stock solution of SM(PEG)_12_ in DMSO was prepared. The amine-targeted reaction (towards the -NH_2_ group of AB-NTA) and sulfhydryl-targeted reaction (towards the –HS groups on the functionalised AFM probes) were accomplished simultaneously in PBS buffer of pH 7.4. To achieve that, the AB-NTA was diluted in PBS buffer of pH 7.4 to a final concentration of 0.3 mM and SM(PEG)_12_ solution was added to final concentration of 1 mM. The AFM probes were incubated in that solution for 2 h. After the reaction was accomplished, the functionalised AFM probes were very gently washed four times and stored in 10 mM Tris pH 7.4 buffer. The presence of Tris–buffer at this step helps to passivate the non-reacted ester groups on the PEG-cross-linker. As a result, the non-specific interaction in our experiments is greatly reduced. The final step was to attach the RC–LH1–PufX complex to the AFM probes. This was achieved by incubating the functionalised AFM probes in 65 nM solution of the protein in 10 mM HEPES of pH 7.4, 250 mM KCl, 0.59 mM β-DDM for 15 min and then very gently washing the probes (4 times) in 10 mM HEPES of pH 7.4, 250 mM KCl, 0.59 mM β-DDM buffer and storing them in 10 mM HEPES of pH 7.4, 45 mM KCl buffer for further use. In parallel with the AFM probes, SiO*_x_* substrates were functionalised exactly in the same way, and at the same time as the AFM probes, which aided assessment of the final surface density of the protein molecules attached to the AFM probes. By varying the concentrations of the SM(PEG)_12_ cross-linker and the protein (in the final step), we adjusted the surface density of protein molecules to ∼200–500 molecules per 1 µm^2^. This roughly corresponds to ∼0.5–1 protein molecule attached to the active area on the apex of the AFM probe. The active area is defined as the part of the apex of the tip where the attached protein molecules can be brought into contact with the proteins on the surface, nominally ∼1500–2000 nm^2^ for the functionalised probes.

### Protein immobilisation and patterning on surfaces

The cytochrome *c*_2_-His_6_ molecules were immobilised onto Ni^2+^-NTA-functionalised SiO*_x_* substrates via their His_6_-tag. The Ni^2+^-NTA functionality was introduced to the previously mercapto-functionalised substrates by attaching the AB-NTA molecule to the –HS groups on the functionalised substrates using succinimidyl-4-(*N*-maleimidomethyl)cyclohexane-1-carboxylate (SMCC, Pierce Biotechnology) — a short (0.9-nm spacer arm) heterobifunctional cross-linker. A 400 mM stock solution of SMCC in DMSO was prepared, and the mercapto-functionalised SiO*_x_* substrates were incubated for 2 h in PBS buffer of pH 7.4 supplemented with AB-NTA, SMCC and NiSO_4_to final concentrations of 5, 20 and 70 mM, respectively. After extensive washing in PBS buffer of pH 7.4, the functionalised substrates were incubated with 15 µM solution of cytochrome *c*_2_-His_6_ for 10 min and then extensively washed in 10 mM HEPES of pH 7.4, 250 mM NaCl, 1 mM LDAO buffer to remove the physisorbed protein. Next, the samples were washed in 10 mM HEPES of pH 7.4, 45 mM KCl buffer and imaged immediately.

Alternatively, a patterned sample was prepared with alternating micrometer-size linear arrays of His_6_-CFP and cytochrome *c*_2_-His_6_ (Supplementary Figure S1). The pattern was achieved by micro-contact printing (µCP) of His_6_-CFP molecules on Ni^2+^-NTA-functionalised SiO*_x_* substrate with subsequent backfilling of the gaps between the His_6_-CFP lines and cytochrome *c*_2_-His_6_. For the µCP, a PDMS stamp was prepared from thermally cross-linked Sylgard 184 (mixing ratio, 1:5) templated against a TGZ04 calibration standard (Mikromasch). After the curing, the PDMS stamp was soaked for 16 h in hexane (Sigma–Aldrich) to remove the non-cross-linked material and dried in pure nitrogen. Next, the stamp surface was oxidised in a UV/Ozone cleaner, and 70 µM aqueous solution of His_6_-CFP was immediately applied onto the stamp for 20 min. Then, the stamp surface was blotted/blown dry and the stamp was applied onto the Ni^2+^-NTA-functionalised SiOx substrate for 20 min. After gently removing the stamp, the patterned substrate was incubated with 15 µM solution of cytochrome *c*_2_-His_6_ for 10 min in order to achieve the backfilling of the pattern. Then, the samples were washed in 10 mM HEPES of pH 7.4, 45 mM KCl buffer, and the quality of the pattern was assessed by fluorescent optical microscopy.

*AFM measurements:* All AFM measurements were performed with a Multimode 8 instrument equipped with a NanoScope V (Bruker) controller. NanoScope (v 8.15) software (Bruker) was used for data collection. Force–distance measurements were performed in imaging buffer (10 mM HEPES of pH 7.4, 45 mM KCl) at room temperature using MSNL (cantilever E) probes. The ramp size was 250 nm with a constant approach velocity of 500 nm s^−1^, the dwell time (i.e. the interval between approach and retraction) was set equal to zero and the retract velocity varied in the range 200–3000 nm s^−1^. The contact force was kept at a low value, ∼100 pN. The spring constant for each cantilever was obtained using the built-in cantilever calibration (thermal method) in the NanoScope software; the obtained spring constants for the cantilevers used for dynamic force spectroscopy (DFS) were in the range 0.082–0.114 N m^−1^. During all force spectroscopy measurements (with the exception of the dark control measurements), the sample and the AFM probe were illuminated from a white light source through an optical fibre (Fiber-Lite MI-150, Dolan-Jener) and the power density of the illumination at the sample surface was measured with Newport 842-PE (Newport Corp.) power meter. This illumination allowed for the repeated photo-oxidation of the RC–LH1–PufX protein attached to the AFM probe after each ET interaction with the cytochrome *c*_2_-His_6_ proteins on the sample surface. Before starting the measurements, the cytochrome *c*_2_-His_6_ proteins on the surface were pre-reduced by incubation in reducing buffer (imaging buffer supplemented with 1 mM sodium dithionite) with subsequent wash in imaging buffer. After recording a short series of force–distance curves, the samples were washed again in reducing buffer, washed in imaging buffer and placed in the AFM to record next series of force–distance curves until sufficiently large data sets were recorded for each sample.

*PeakForce QNM measurements* were performed under the same conditions as described above with BL-AC40TS probes (Olympus). The spring constants measured for these cantilevers were in the range 0.062–0.174 N m^−1^. The Z-modulation amplitude was adjusted to values in the range 20–25 nm to allow enough tip-sample separation in order to fully stretch the PEG linker molecule on the AFM tip and to separate the RC–LH1–PufX from the cytochrome *c*_2_-His_6_ molecules on the surface during each ramp cycle. The contact tip-sample force was kept in the range 60–100 pN, and the imaging rate was adjusted (depending on the scan size and pixel density of the scan) in a way that ensured one force–distance curve recorded per image pixel. Three different modulation frequencies were used (0.5, 1 and 2 kHz) to record quantitative nanomechanical mapping (QNM) data, effectively resulting in three different loading rates for the QNM force spectroscopy data. The QNM force–distance curves were recorded by using the high-speed data capture (HSDC) capability of the NanoScope software which allows simultaneous recording of cantilever deflection error vs. Z-position and Z-position vs. time data over selected part of the AFM scan. It is worth noting that because the Z-modulation amplitude is a sine function, the approach and retract velocities during the PeakForce imaging are not constant. Nevertheless, it is possible to obtain the instantaneous tip velocity at any point of the force–distance curve from the Z-position vs. time data acquired via the HSDC. To ensure stable specific interaction between the proteins attached to the sample surface and their redox partner on the AFM probe after acquiring two to three AFM scans, the samples were consecutively washed in reducing imaging buffer and re-imaged again.

For the control experiments, the docking site of the RC–LH1–PufX protein on the AFM probe was blocked by injection of a 10-fold molar excess of free pre-reduced cytochrome *c*_2_-His_6_ directly into the AFM imaging cell. Alternatively, the RC–LH1–PufX protein was chemically oxidised (treated with 0.8 mM potassium ferricyanide solution) and then washed in imaging buffer in the dark. In this case, the control AFM measurements were conducted in a dark box with the only illumination to the sample and the AFM probe being the 639-nm laser used in the optical lever detection system for the AFM.

*Data analysis:* All the AFM data were analysed using Gwyddion v 2.51 (open source software covered by GNU general public licence, www.gwyddion.net), Nanoscope Analysis v 1.5 (Bruker), PUNIAS v1r21 (www.punias.voila.net) and OriginPro 2016 (OriginLab Corp.) software. Gwyddion and Nanoscope analysis were used for image processing and analysis. Nanoscope analysis was also used for the extraction and analysis of the QNM force spectroscopy data. PUNIAS and OriginPro were used for the statistical analysis of all the force spectroscopy data, and OriginPro was also used for all the calculations and fittings.

Data reduction (positive identification of specific rupture events) was based on two criteria: rupture events occurring at tip-sample separation in the range 5–15 nm, and a satisfactory fit of a WLC model to the characteristic non-linear part of the retract curve preceding the rupture event. The effective loading rate values were determined from the slope of the force curve immediately before (≤1 nm) the position of the rupture point. The most probable values for the rupture force and the loading rate were obtained from the maximum of the Gaussian fit to the force and loading rate distribution combined in a statistical histogram. Normally, the rupture forces and loading rates of a few hundred rupture events were compiled in force or loading rate distribution histograms. In the case of QNM force spectroscopy data, each recorded force–distance curve contains 256 data points (128 points for the approach part of the curve and 128 points for the retract part of the curve) which is better or equal to the number of data points available for a force–distance curve recorded in a conventional force-volume imaging mode [35], thus allowing us to positively identify the rupture events.

## Results

### Dynamic force spectroscopy of the interaction between cytochrome *c*_2_ and the RC–LH1–PufX membrane protein complex

**Figure 2.**
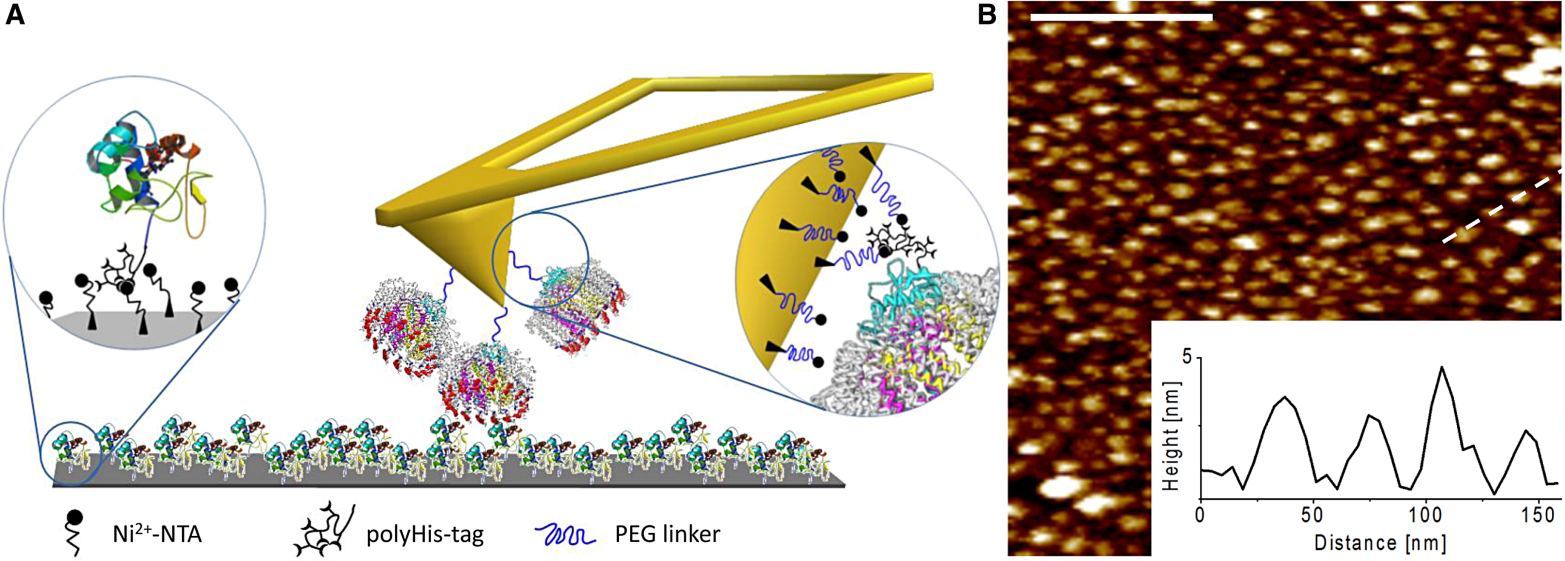
Surface attachment of RC–LH1–PufX complexes and cytochrome *c*_2_ molecules. (**A**) Schematic representation of RC–LH1–PufX complexes immobilized on the AFM probe and sample substrate, comprising closely packed cytochrome *c*_2_ molecules. (**B**) AFM topography image (in liquid) of closely packed individual cytochrome *c*_2_ molecules on the SiO*_x_* substrate. The dashed white line indicates the height profile taken across four immobilised cytochrome *c*_2_ molecules, and the scale bar is 200 nm. The surface density is ∼1000–1500 molecules per µm^2^. The height profile in the inset shows an average height of the molecules of ∼3 nm while the average lateral size (FWHM) is in the range 13–20 nm.

To investigate and quantify the interaction forces between the cytochrome *c*_2_ electron carrier and the monomeric His_12_–RC–LH1–PufX (henceforth RC–LH1–PufX) core complex (Figure 1C,D), these proteins were attached to a silicon oxide (SiO*_x_*) substrate and an AFM probe, respectively. RC–LH1–PufX core complexes, each bearing a C-terminal His_12_-tag on the RC H-subunit, were attached to SiN AFM tips via silane monolayer chemistry and a polymer linker molecule (PEG_12_) terminated with Ni^2+^-NTA groups. Cytochrome *c*_2_ proteins, each carrying a C-terminal His_6_-tag, were immobilised on a SiOx substrate functionalised, again via silane monolayer chemistry, with Ni^2+^-NTA (Figure 2A). The strategy of using His-tags with a long flexible spacer ensures that the RC–LH1–PufX complex molecules on the AFM tip, and cytochrome *c*_2_ molecule simmobilised on the surface, are free to move and orient, favouring complex formation [31,36]. The coverage of the immobilised cytochrome *c*_2_-His_6_ molecules on the functionalised SiOx substrate was assessed by PeakForce Tapping™ AFM in imaging buffer (10 mM HEPES of pH 7.4 and 45 mM KCl) with a blank (non-functionalised) probe, revealing a surface density in the range of 1000–1500 cytochrome *c*_2_-His_6_ molecules per µm^2^ (Figure 2B). The average height of the molecules was measured to be ∼3.3 nm, while the average lateral size (FWHM) is in the range 15–20 nm (the inset in Figure 2B), consistent with the expected size of the cytochrome *c*_2_-His_6_ molecule and taking into account increased lateral dimensions due to the geometrical tip convolution effects.

**Figure 3.**
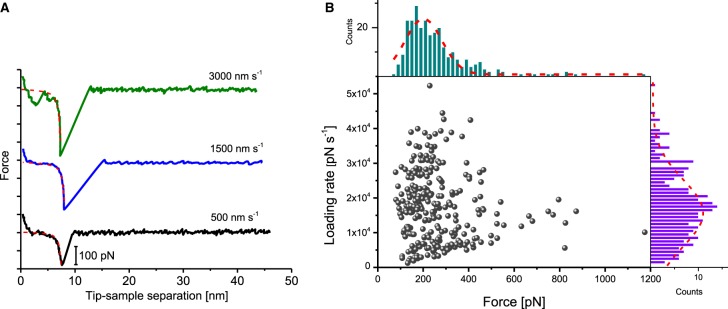
Dynamic force spectroscopy of the interaction forces between cytochrome *c*_2_ and the RC–LH1–PufX membrane protein complex. (**A**) Typical force-distance curves recorded upon the retraction of the functionalised probe from the surface at three different velocities (500, 1500 and 3000 nm s^−1^). All three curves clearly display a characteristic unbinding event (with worm-like chain (WLC) – see Supplementary Information) fits to the curves indicated with red dashed lines) with rupture lengths in the range 7–8 nm and rupture forces of ∼185, 272 and 375 pN, respectively. For clarity, the curves are offset along the *Y*-axis, and the scale bar for *Y*-axis is 100 pN. (**B**) Distribution of the unbinding force vs. loading rate with corresponding histograms and Gaussian fits for one of the three data sets, corresponding to data obtained with a retraction velocity of 500 nm s^−1^.

**Figure 4.**
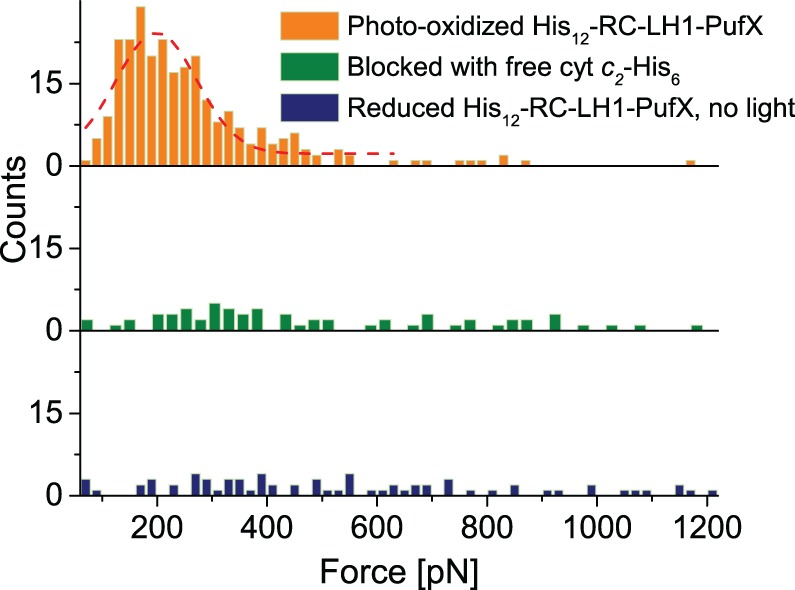
Distribution of the interaction forces during control experiments verifying the specificity of the unbinding events. Force distribution (most probable force obtained from the Gaussian fit, dashed line) for the specific unbinding between RC–LH1–PufX and the cytochrome *c*_2_–His_6_ (top), with two negative controls — blocking the RC docking site with free cytochrome *c*_2_-His_6_ (middle) and dark measurements preventing photooxidation of tip-attached RC–LH1–PufX complexes (bottom).

After finding an area of the sample surface with uniform coverage of cytochrome *c*_2_-His_6_, the AFM probe for topology imaging was replaced with a probe functionalised with RC–LH1–PufX complexes for DFS in contact mode, again in imaging buffer. Many data sets (each consisting of ∼1000 force–distance curves) were recorded over different locations on the sample and at different probe retraction velocities. Each data set was analysed to evaluate the unbinding probability as well as the most probable unbinding force and most probable loading rate (see Materials and methods). Figure 3B shows a plot of the distribution of rupture forces versus the loading rate for one data set (retraction velocity, 500 nm s^−1^), with the corresponding histograms (and Gaussian fits) along the axes measured from 346 unbinding events over 1703 force–distance curves, a binding probability of 20.3%. From the Gaussian fits of the force and loading rate histograms, we estimate the most probable unbinding force to be 198.9 ± 6.7 pN and the most probable loading rate to be 15 287 ± 1139 pN s^−1^ (unless stated otherwise, all values are given as mean ± s.e.). The results for all the different loading rates are summarised in Table 1. In order to verify the specificity of the observed unbinding events, we performed control experiments to test the inhibition of the specific binding between the RC–LH1–PufX and cytochrome *c*_2_-His_6_ proteins. The first control was to block the docking site on the photo-oxidised, tip-bound RC–LH1–PufX with free pre-reduced cytochrome *c*_2_-His_6_ injected into the AFM liquid cell at a final concentration of 3 µM (an order of magnitude greater than the *K*_D_ of ∼0.3 µM [22]). After incubation with free cytochrome *c*_2_-His_6_ for ∼10 min, new data were recorded at the same retraction velocity. Analysis of the data obtained after blocking revealed a weak peak at ∼ 280 pN in the unbinding force histogram, but the probability for binding decreased by a factor of 5 (Figure 4). Since the RC–LH1–PufX has to be in an oxidised state in order to accept an electron from the reduced cytochrome *c*_2_-His_6_, a second control experiment was performed by chemically reducing the RC–LH1–PufX complex while conducting the force spectroscopy measurements in the dark to prevent RC photo-oxidation. These conditions should effectively switch off the specific interaction between the cytochrome *c*_2_-His_6_ and the RC–LH1–PufX proteins. Analysis of the force data recorded under reducing conditions in the dark revealed that the binding probability was further decreased with no prominent peak observed in the force histogram. The low, residual binding activity observed, even under these very effective blocking conditions, could reflect the forced, non-specific interaction between the two molecules, brought together by the descent of the tip to the surface.

**Table 1:** Summary of the most probable loading rates with the corresponding most probable rupture forces obtained from the DFS and PF-QNM measurements

Type of force microscopy	Unbinding force (pN)	Loading rate (pN s^−1^)
DFS	112 ± 3.8	5301 ± 380
	198 ± 3.8	15 287 ± 1139
	261 ± 18.4	22 693 ± 1043
	362 ± 22	55 426 ± 1800
PF-QNM	73 ± 42	1.029 × 10^6^ ± 1.76 × 10^5^
	565 ± 53	2.95 × 10^6^ ± 4.67 × 10^5^
	887 ± 67	8.938 × 10^6^ ± 6.67 × 10^5^

To verify whether our experimental data had included non-specific interactions, we brought a functionalised AFM probe (with RC–LH1–PufX attached to the tip) into contact with a blank (no cytochrome *c*_2_-His_6_ attached) Ni^2+^-NTA SiO*_x_* substrate and found that such interactions took place with only a very low probability (Supplementary Figure S2).

### Surface mapping and quantification of cytochrome c_2_–RC interactions using PeakForce QNM single-molecule force spectroscopy

The PF-QNM technique is based on the PeakForce Tapping™ technology developed by Bruker Inc., which generates nanoscale images where each pixel comprises force–distance curves that reflect the mechanical properties of the sample such as elasticity, deformation and adhesion, as well as topography [31,37]. Unlike dynamic force spectroscopy in Figures 3 and 4, PF-QNM can identify a binding event that involves a particular molecule on the surface, enabling the mapping of complexes in a membrane, for example, the distribution of cytochrome *b*_6_*f* complexes in the grana regions of plant thylakoids [37].

**Figure 5.**
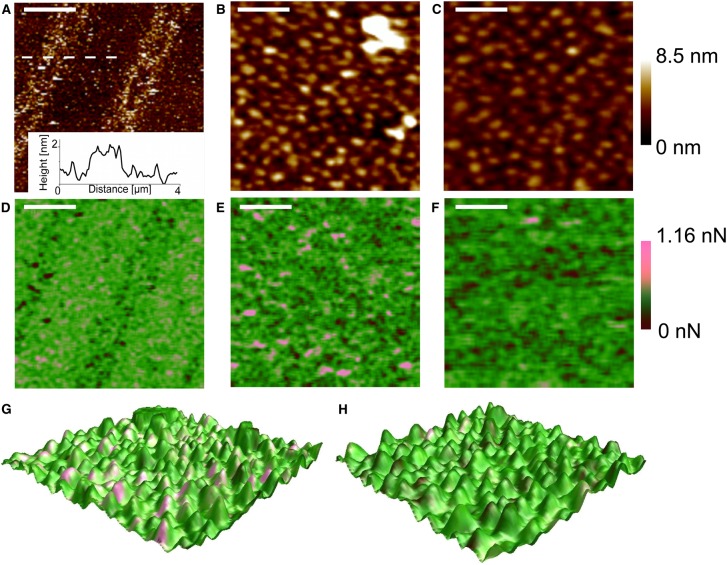
PeakForce-QNM functional imaging of alternating linear arrays of cytochrome *c*_2_ and CFP. (**A**) Large-scale topography of the CFP/His-cytochrome *c*_2_ patterned sample (inset showing the height difference between the two proteins, using the height profile corresponding to the white dashed line in the main panel; the scale bar is 2 µm. (**B**) Molecular scale topography of a region of the substrate with immobilised cytochrome *c*_2_ molecules; the scale bar is 80 nm. (**C**) The same sample as in (**B**) but recorded in the presence of excess free cytochrome *c*_2_-His_6_; the scale bar is 80 nm. (**D**) Adhesion image corresponding to the topography in (**A**); the pink colour corresponds to the specific events (high unbinding force), while the green colour (and its darker shades) correspond to the non-specific interactions. The colour bar on the right shows the adhesion scale, from 0 to 1.16 nN. (**E**) Adhesion image corresponding to the topography in (**B**); (**F**) adhesion image corresponding to the topography in (**C**); (**G** and **H**) 3D composite images (topography combined with adhesion skin) of the specific unbinding events from (**E**) and (**F**).

We used PF-QNM to follow the unbinding events between the RC–LH1–PufX complex and its cytochrome partner whilst simultaneously imaging the surface distribution of cytochrome molecules. To test the ability of the technique to discriminate between the desired target and a similar-sized protein, we prepared samples where cytochrome *c*_2_-His_6_ and the His-tagged cyan fluorescent protein (His_6_-CFP) were patterned on the functionalised SiO*_x_* substrate in linear, micrometre-size alternating arrays (see Materials and methods section). A topography image (Figure 5A) was recorded in imaging buffer with a RC–LH1–PufX-functionalised AFM probe, using a modulation frequency of 1 kHz. A pattern can be clearly seen, arising from the small difference in the height, ∼1.3 nm (inset in Figure 5A), between the two proteins attached to the surface; the higher and narrower parts of the pattern correspond to the His_6_-CFP linear arrays, and the lower, wider regions are filled with cytochrome *c*_2_-His_6_. Simultaneously with the topography, an adhesion image was recorded (Figure 5D); the regions with immobilised cytochrome *c*_2_-His_6_ are characterised by high-adhesion (or high unbinding force) events, indicated in pink (see quantitative scale, 0–1.16 nN, on the right), and low adhesion (unbinding force) events are found in those parts of the sample (black/dark green) with patterned His_6_-CFP, for which the tip-bound RC–LH1–PufX complex has no affinity. Thus, functional AFM imaging using a tip derivatised with RC–LH1–PufX complexes readily identifies cytochrome *c*_2_-patterned areas on the basis of the recognition forces associated with docking of cytochrome *c*_2_ onto its binding site on the RC–LH1–PufX complex. Upon zooming-in to one of the cytochrome *c*_2_-His_6_ regions, individual cytochrome molecules are seen in the topographic image (Figure 5B). Their average height is ∼3.7 nm, and their lateral size is again in the range 19–23 nm, consistent with observations of the samples covered with a homogeneous monolayer of cytochrome *c*_2_-His_6_ in Figure 2B. The corresponding adhesion image, Figure 5E, clearly shows individual unbinding (high-adhesion force) events, although only 42 of the 126 cytochrome *c*_2_-His_6_ molecules in the topography image interacted with the RC-functionalised AFM tip. For a molecule-by-molecule comparison, the topology and adhesion images were combined in a 3D image (Figure 5G), where the profile represents the sample topography and the colour-coding indicates the relative strength of the adhesion (or unbinding) forces — the pink colour corresponds to the specific events (high unbinding force), while the green colour (and its darker shades) corresponds to the non-specific interactions. In this composite image, the specific unbinding events correlate well with the topologies of individual surface-attached cytochrome *c*_2_-His_6_ molecules although the high-adhesion events are slightly offset from the centres of the molecules, as also seen in an earlier study [31].

As a control experiment, and as for the dynamic force experiment in Figure 4, the specific RC–cytochrome *c*_2_ interaction was blocked by an excess of pre-reduced cytochrome *c*_2_-His_6_, injected into the AFM liquid cell at a final concentration of 3 µM. Figure 5C shows the retained topology, and Figure 5F,H shows that the high unbinding force events almost completely disappear from the adhesion image (compare with Figure 5E,G). Thus, the high-adhesion force events we observed in the adhesion images are associated with the specific interaction between the tip-bound RC–LH1–PufX and surface-attached cytochrome *c*_2_-His_6_ proteins.

**Figure 6.**
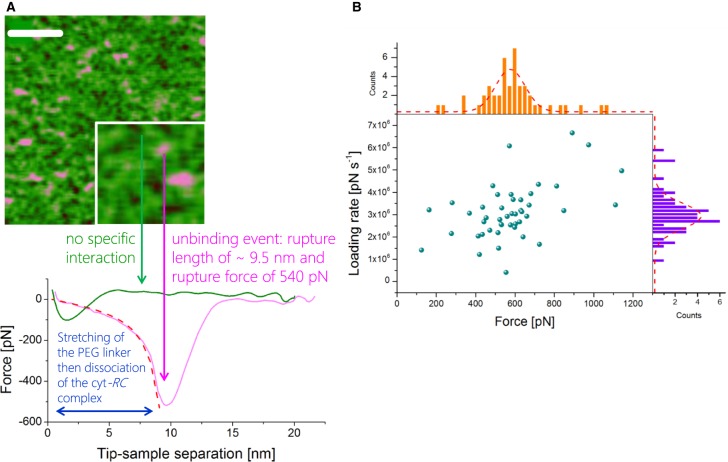
Quantitative analysis of cytochrome *c*_2_–RC interactions by PF-QNM force spectroscopy. (**A**) Examples of force-distance curves extracted from the PF-QNM data corresponding to Figure 5E. The green curve shows no specific interaction. The pink curve, recorded over the spot indicated, corresponds to an unbinding event that reflects stretching of the linker to a length of ∼9.5 nm and then rupture with a force of 540 pN. The red dashed line shows a WLC fit (see Supplementary Information) to the curve. (**B**) Distribution of the unbinding forces vs. loading rates for a total of 46 events with corresponding histograms and Gaussian fits.

In addition to spatially mapping intermolecular interactions, PF-QNM also allows their quantification following extraction and analysis of the force-distance data obtained from a selected area of the image. Examples of force–distance curves extracted from the PF-QNM data in Figure 5E are shown in Figure 6A. The green curve is recorded over the dark spot indicated in Figure 6A, which corresponds to no specific interaction, whereas the pink curve, recorded over the pink spot in the same figure, corresponds to an unbinding event with a rupture length of ∼ 9.5 nm and a rupture force of 540 pN. By calculating the slope of the curve just prior to the rupture event and multiplying by the measured instantaneous velocity of the probe (132.8 µm s^−1^ in this case), one can obtain the effective loading rate for the rupture event. We were able to extract and analyse (using the same algorithm as for the DFS data) 46 force–distance curves recorded over many ‘high-adhesion’ spots acquired at 1-kHz modulation frequency; the distribution of the rupture forces versus the loading rate and the corresponding histograms (and Gaussian fits) is presented in Figure 6B. The most probable unbinding force was found to be 565 ± 53 pN and the most probable loading rate of 2.95 × 10^6^ ± 4.67 × 10^5^ pN s^−1^, corresponding to a modulation frequency of 1 kHz. The loading rate can be varied by manipulation of the modulation frequency during the PF-QNM data acquisition; we recorded adhesion images displaying specific high unbinding force events at modulation frequencies of 0.5, 1 and 2 kHz and analysed the data as described in Materials and methods section; the results of all three frequencies are summarised in Table 1.

### Comparative analysis of measurements from dynamic force spectroscopy and PF-QNM force spectroscopy

In principle, it is possible to determine the dissociation rate of a bond from loading rate-dependent measurements of the unbinding force. The relationship between the experimentally measured bond rupture forces and the interaction potential is described by a kinetic model first proposed by Bell [38] and then developed by Evans and co-workers [39,40]. Exponential amplification of the bond dissociation rate, *k*_off_, by an external loading force produces a characteristic proportionality of the rupture force, F*, to the logarithm of the loading rate, $R_{0}$*:*

1 $$F^{*}=\frac{k_{B}T}{x_{\beta }}ln\left(\frac{R_{o}x_{\beta }}{k_{\mathrm{off}}k_{B}T}\right),$$

where $k_{B}T$ is thermal energy and $x_{\beta }$ is the characteristic bond length.

**Figure 7.**
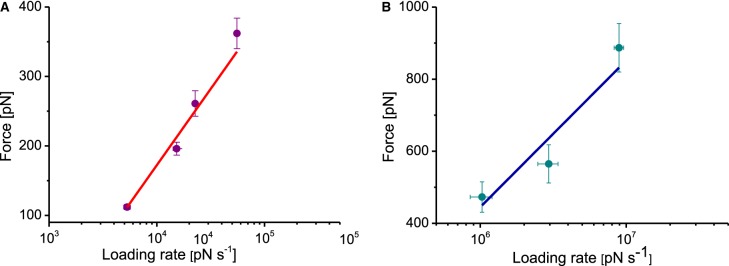
Comparison of dynamic force spectra for DFS and PF-QNM data. (**A**) Rupture force dependence on the logarithm of the loading rate for DFS data, and (**B**) for PF-QNM data. The linear fits of eqn 1 (red and blue lines) yield off-rate constants, *k*_off_, of 7.5 and 198 s^−1^, respectively.

The unbinding force data from the DFS and PF-QNM measurements are plotted versus loading rate in Figure 7A,B, respectively. Each data set shows a linear dependence on the logarithm of the loading rate, with the slope of each line related to the characteristic bond length and the intercept related to the bond length and zero force lifetimes. The two free parameters of the above relationship, $x_{\beta }$ and *k*_off_, were obtained for both DFS and PF-QNM data by fitting eqn (1) to the data points in each plot. For the DFS data (Figure 7A), we obtained $x_{\beta }$ = 0.02 nm and *k*_off_ = 7.5 s^−1^, whereas the values for the PF-QNM data (Figure 7B) were $x_{\beta }$ = 0.01 nm and *k*_off_ = 198 s^−1^.

## Discussion

### Comparison of DFS and PF-QNM approaches for force spectroscopy of the cytochrome *c*_2_–RC ET complex

Here, we report a single-molecule study of the transient association of interaction of cytochrome *c*_2_ with the RC–LH1–PufX complex, using two force spectroscopy approaches to measure the forces required to disrupt the post-ET complex. The type of protein–protein association found in the cytochrome *c*_2_–RC complex is widely found in bioenergetic systems, which often require the docking of an extrinsic electron donor with a membrane-bound acceptor complex. The native environment of the cytochrome and RC proteins is depicted in Figure 1; here, we have created an *in vitro* system where the two proteins are brought together in a controlled fashion; one is attached to a planar substrate, and the other to a retractable AFM probe. The tethering of each type of molecule, to either the surface (cytochrome *c*_2_) or the AFM probe (RC–LH1–PufX complex), relies on His-Ni^2+^-NTA coordination bonds, which can sustain significant loading forces for prolonged periods of time [41–44]. The rupture forces for the His-Ni^2+^-NTA bridge have been measured to be in the range 50–500 pN for loading rates in the range 3000–68 000 pN s^−1^ [45–49]. In our DFS experiment, the applied loading rates were in the range 5000–56 000 pN s^−1^ and the measured unbinding forces in the range 112–362 pN. These values were entirely within the range of forces sustained by the His-Ni^2+^-NTA bridge without rupture. It has also been shown that longer polyhistidine tags (6 and more His residues), especially when facilitated by PEG linker molecules, can stably bind to pairs or triples of Ni^2+^-NTA groups, further increasing the forces that can be applied during the DFS measurements [50].

There are charged Lys and Arg residues at the C-termini of the LH1 transmembrane helices on the periplasmic (lumenal) side of the chromatophore membrane. Thus, the cytochrome *c*_2_ docking site on the RC is surrounded by a ring of positive charges that could restrict the possible *in vivo* pathways for association and dissociation between the two proteins. It is possible that, when leaving the docking site on the RC, the positively charged cyt *c*_2_ has to migrate out of plane (perpendicular to the membrane surface), repelled by the charged residues on LH1. This probable pathway coincides with the pulling trajectory in our experiments (both DFS and PF-QNM imaging). The configuration of our experiment has RC–His_12_–LH1–PufX molecules attached to the AFM probe and cytochrome *c*_2_-His_6_ proteins tethered to the surface; this arrangement ensures that during the image acquisition scan, as the probe moves from pixel to pixel and acquires a force–distance curve at each pixel, a photo-oxidised RC–LH1–PufX on the probe can interact with (and accept an electron from) a ‘fresh’ reduced cytochrome *c*_2_-His_6_ molecule. Another consideration in our experimental set-up is the fact that purified RC–LH1–PufX complexes were found to retain 25–30% of the endogenous quinone acceptor pool [51]. This quinone pool stores the electrons resulting from the photo-oxidation of the primary donor, thus maintaining the turnover of the ET cycle during image acquisition.

The unbinding forces measured during the PF-QNM experiments are in good agreement with theoretical work on the interaction pathways of the cytochrome *c*_2_–RC–LH1–PufX ET complex, which estimates the forces acting during the dissociation process to be in the range 600–1000 pN [29,30]. In addition, previous DFS experiments on a variety of other protein–protein interactions report forces in the range 450–1300 pN [52–55]. This is a good indication that the values from our PF-QNM experiments, although high, are not unusual for protein–protein interactions. The fact that almost no interactions could be measured when the RC–LH1–PufX complex was chemically reduced in the dark, preventing the formation of the photo-oxidised RC electron acceptor state and ET from reduced cytochrome *c*_2_-His_6_, indicates that the unbinding events we have observed and analysed arise from formation of the ET complex between the cytochrome *c*_2_ and RC–LH1–PufX proteins. Consistent with this proposal, we observed a significant drop in the binding probability, from ∼20% to ∼4%, upon blocking the docking site on the RC–LH1–PufX complex with free cytochrome *c*_2_-His_6_. The residual binding probability in the blocking control likely arises from the dynamic nature of binding between the RC–LH1–PufX complex on the tip and the free cytochrome *c*_2_-His_6_ in solution, which would leave the binding site on the RC unblocked for short periods, and free to associate with surface-attached cytochrome *c*_2_. It is worth noting that the binding probability of 20.3% in our experiment is comparable with 18% found for the cytochrome c551/Azurin pair [56] and 25% for the biotin/streptavidin pair [57].

It is well established that the cytochrome *c*_2_ to RC ET occurs on a sub-millisecond timescale with fast (∼1 μs) and slower (50–200 μs) phases [18,59]. The half-life of the bound state will be of the order of hundreds of µs, with an off-rate constant *k*_off_, corresponding to the dissociation of oxidised cytochrome *c*_2_ from newly reduced RCs, of ∼5 × 10^3 ^s^−1^ (at ionic strength similar to our experimental conditions) [27]. The off-rate constant *k*_off_ value of 7.5 s^−1^ we obtained from DFS measurements is in good agreement with that of the force spectroscopy data obtained for the cytochrome *c* 551–azurin ET complex [56,59], but is clearly different from the *k*_off_ value for unbinding of cytochrome *c*_2_[oxidised] from the RC obtained by spectroscopic ensemble-averaged methods. The most plausible reason for this discrepancy is the mismatch between the half-life time of the bound state of the ET complex and the loading time, that is, the time required to fully stretch the PEG-linker and to finally separate the molecules upon retraction of the AFM probe. In our DFS experiments, the dwell time on the surface time is in the range 2–10 ms (depending on the retract velocity), at least an order of magnitude larger than that of the half-life time of the bound state. In such circumstances, the molecules have time to unbind spontaneously (and possibly even re-bind and unbind again) prior to the full stretching of the polymer linker and the application of the external force. Thus, a pulling rate that is not fast enough to separate the molecules when they are in an initially bound state reflects apparent values rather than the actual binding probability and life-time of the bond.

In contrast, the PF-QNM force measurements yield a *k*_off_ value of 198 s^−1^, which is much higher than the value obtained from DFS and somewhat closer to the published data for the bacterial cytochrome *c*_2_–RC complex [27]. The PF-QNM force measurements show a higher binding probability (33%), compared with DFS (20%); this demonstrates the ability of PF-QNM quantitative imaging to actually measure forces at substantially (up to 2 orders of magnitude) higher loading rates and with a tip-sample contact time in the range 70–240 µs (depending on the modulation frequency), i.e. lower than the half-life time of the bound state of the ET complex. The pulling rates are sufficiently fast to access the transient bound state and to investigate the dissociation kinetics in our model system on a faster, and therefore more biologically relevant, timescale compared with those of DFS.

### Complementary redox states stabilise the ET and post-ET complexes

Complementarity of redox states, namely cytochrome *c*_2_[red]–RC[ox], is required to form an ET complex, and cytochrome *c*_2_[red]–RC[red] is disfavoured. We assume that that, in the case of the DFS and PF-QNM measurements, the bound complex is sufficiently long-lived to allow rapid ET and generation of a post-ET cytochrome *c*_2_[ox]–RC[red] state. Thus, it is this state that is disrupted by the pulling forces even though one might have expected that, following ET, the two reactants would simply separate and diffuse apart with no force required to separate them. The present results, taken together with an earlier AFM study [31], and an all-atom SMD simulation of the RC–cytochrome *c*_2_ complex [30], show that the redox states of the cytochrome *c*_2_ haem cofactor and RC BChl_2_ ‘special pair’ cofactors play a crucial role in establishing a productive ET complex, in addition to the electrostatic, hydrophobic, hydrogen bonding and cation–π interactions at the RC–cytochrome *c*_2_ interface [14,15]. The cofactor redox states appear to be the major influence on complex formation, over-riding the ‘ground state’ electrostatics of the RC–cytochrome *c*_2_ pair; in this way, unproductive encounters between, for example, cytochrome *c*_2_[red]–RC[red] and cytochrome *c*_2_[ox]–RC[ox] are avoided. The formation of cytochrome *c*_2_[red]–RC[ox] is therefore favourable, leading to ET and resetting the RC for another round of photochemistry and cyclic ET. However, another consequence of favourable complementarity of redox states is the persistence of the post-ET state cytochrome *c*_2_[ox]–RC[red], measured by our AFM approach. The fact that a force of 540 pN is required to separate the cytochrome *c*_2_[ox]–RC[red] product state has an interesting parallel with the docking/undocking of cytochrome *c*_2_[ox]/[red] onto cyt*bc*_1_[red]/[ox], which is the other step in the cyclic ET found in this photosynthetic bacterium (see Figure 1). A molecular dynamics study of this process also showed redox state-dependent binding, and it identified a molecular switch, involving Lys99 of cytochrome *c*_2_, which responds to the redox state of the nearby haem and forms an association with Glu95 of cytochrome *c*_1_ on the periplasmic face of the cyt*bc*_1_ complex [60]. Similarly, an MD study of the cytochrome *c*_2_–RC association invokes Lys97 and Lys99 of cytochrome *c*_2_ in the docking/undocking process [30]; as cytochrome *c*_2_ was pulled from the RC surface, the simulation found a strengthening salt bridge between LysC97 and AspL261 of the RC-L subunit and that significant electrostatic interactions persist for 10 Å separations of cytochrome *c*_2_[ox] and the RC [30].

### The cytochrome *c*_2_–RC ET complex in the context of the cyclic ET pathway in bacterial photosynthesis

The persistence of the cytochrome *c*_2_[ox]–RC[red] ‘product’ state on a timescale of many milliseconds, a duration orders of magnitude larger than the cytochrome *c*_2_ → RC ET time, have been interpreted as a form of ‘product inhibition’, first noted by Moser and Dutton [19] and more recently by Gerencsér et al. [27]. However, consideration of the intact cyclic ET system housed by an intact chromatophore vesicle (see Figure 1) provides a wider context for thinking about decay of the cytochrome *c*_2_[ox]–RC[red] ‘product’ state and return of cytochrome *c*_2_[ox] to the cytochrome *bc*_1_ complex, where it is reduced [58]. The energy conversion processes of a complete chromatophore vesicle consisting of more than hundred complexes, from ‘photon to ATP’, have been modelled [11]. At a light intensity equivalent to 1–5% of full sunlight, a typical low-light growth regime for *Rba. sphaeroides*, each of the 24 RCs in a chromatophore vesicle absorbs ∼16–78 photons per second [11], which generates an oxidised RC every 13–65 milliseconds. Cyclic ET requires docking of cytochrome *c*_2_[red] onto a photo-oxidised RC, ET, then departure of cytochrome *c*_2_[ox] from RC[red] and diffusion to the cytochrome *bc*_1_ complex. These processes, as well as cytochrome *c*_2_ reduction by the cytochrome *bc*_1_ complex, are accomplished on a timescale of tens of milliseconds. We also note that the system as a whole is limited by quinol turnover at the cytochrome *bc*_1_ complex [11], which is 25 ms [61,62]. Thus, a duration of milliseconds for the cytochrome *c*_2_[ox]–RC[red] ‘product’ state, probed here by PF-QNM, is neither inhibitory nor rate limiting for cyclic ET, and it is compatible with the turnover kinetics of the intact cyclic ET system, at realistic incident light intensities.

### Postscript

Some of this work was presented as part of a Keilin Medal Lecture, in which it was noted that David Keilin, the discoverer of cytochromes [63], had made a series of pivotal discoveries using a microspectroscope that allowed him to visualise cytochrome absorption bands directly and to observe their transient oxidation and reduction reactions in living tissue in real time. Keilin introduced the concept of a respiratory chain and stressed the importance of the spatial orientation of chain components, a remarkable insight. He established the framework for understanding cell respiration, stated as Keilin's greatest contribution to biological chemistry [64]. It was also noted that Keilin found ‘much aesthetic satisfaction in visual spectroscopy and was sorry for those to whom cytochrome is no more than a line drawn by a servo-operated pen’ [65]. Perhaps he had a point and now there are new ways to investigate the properties of cytochromes, as shown here using cytochrome *c*_2_. One would like to think that Keilin, who discovered *c*-type cytochromes [66], would have appreciated the ability of an atomic force microscope to image single cytochrome *c*_2_ molecules and to record their individual ET properties, molecule by molecule. Although we can now visualise, measure and model whole ET assemblies by structural, kinetic and computational methods [11], we continue to marvel at David Keilin's insights into respiration and his extraordinary achievements.

## Abbreviations

AB-NTA: 5-(amino-1-carboxypentyl) iminodiacetic acid
AFM: atomic force microscopy
BChl: bacteriochlorophyll
CFP: cyan fluorescent protein
cyt: cytochrome
DFS: dynamic force spectroscopy
ET: electron transfer
FWHM: full width at half maximum
His: histidine
LDAO: *N*,*N*-dimethyldodecylamine *N*-oxide
LH: light harvesting
MPTMS: 3-mercaptopropyltrimethoxysilane
PF-QNM: PeakForce quantitative nanomechanical mapping
PEG: polyethylene-glycol
*Rba*.: *Rhodobacter*
RC: reaction centre
SiO*_x_*: silicon oxide
SMCC: *N*-maleimidomethyl)cyclohexane-1-carboxylate
SMD: steered molecular dynamics
WLC: worm-like chain
