# Supplementary material for: Dissecting the cytochrome c2–reaction centre interaction in bacterial photosynthesis using single molecule force spectroscopy
Source: Biochem J. 2019 Aug 9;476(15):2173–90. doi: 10.1042/BCJ20170519 (PMC6688529; doi:10.1042/BCJ20170519)
Supplement: Supplementary Figures [file BCJ-476-2173-s1.pdf]

Supplementary Information

1. Micro-patterning of His_6-CFP / cytochrome c_2-His_6 linear arrays.

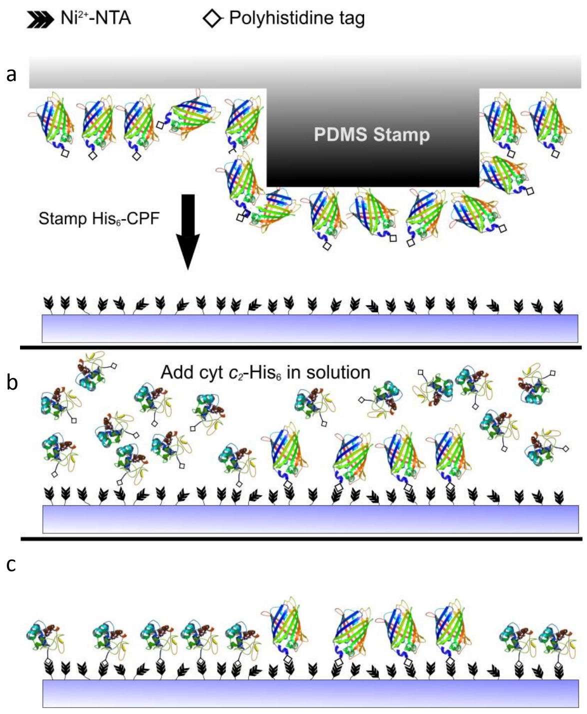

Figure S1. Patterning of cyt c_2-His_6 / His_6-CFP.
(a) – Micro-contact printing of a masking layer of His_6-CFP on a Ni^{2+}-NTA functionalised SiOx substrate. (b) – Filling-in the non-maslked parts of the substrate with cyt c_2-His_6 form solution. (c) – Alternating linear arrays of His_6-CFP and cyt c_2-His_6 formed on the surface.

2. The worm-like chain (WLC) model.

The characteristic stretching of the flexible PEG linker (due to the applied force resulting from the specific binding between the two proteins), preceding the complex dissociation, allows us to clearly discriminate specific unbinding events from non-specific adhesion [1]. The non-linear elastic response of the PEG-linker to the external force can be described by a worm-like chain (WLC) model [2]. The part of the curve immediately prior each rupture event can be fitted (dashed line in Fig. 2A in Results section of the main text) by the equation approximating the WLC model:

$$F(x) = \frac{k_B T}{l_p}\left(\frac{1}{4\left(1-\frac{x}{L_c}\right)^2} - \frac{1}{4} + \frac{x}{L_c}\right), \qquad \text{(S1)}$$

where $F(x)$ is the elastic restoring force of the polymer chain, x is the chain end-to-end distance, $k_B T$ is the thermal energy, l_p is the persistence length of the polymer chain and L_c is the contour

length of the fully extended chain. We used a nonlinear least square fitting method, setting the total contour length L_c equal to the rupture distance and keeping the persistence length l_p as a fitting parameter. From the fitting of the retract curve with equation (S1) we obtained l_p = 0.35 ± 0.022 nm, consistent with the expected persistence length for a PEG chain.[1]

The best fit of eq. (S1) to the part of the curve just prior the rupture event (red dashed line, Figure 6A in Results section of the main text) gives a value for l_p of 0.37 ± 0.048 nm, in satisfactory agreement with the expected persistence length of the PEG linker on the AFM probe. The most probable reason for the deviation of the fit from the recorded data at high forces is due to the non-linear pulling rate of the AFM probe (sine function tip velocity, see the Experimental Section) and the possible attachment of the long His_{12}-tag to more than one linker molecule on the AFM probe.

3. Non-specific interactions between the functionalised AFM probe and a cytochrome-free substrate.

In order to exclude as much as we can the non-specific interactions from our experimental data we studied the interaction between a functionalised AFM probe (with RC-His_{12}-LH1-PufX attached to the tip) and a blank (no cyt c_2-His_6 attached) Ni^{2+}-NTA Si substrate under the same conditions described in the Experimental Section for the DFS and PF-QNM measurements. More than 300 force distance curves were recorded at a retraction velocity of 500 nm s^{-1} (Figure S2a) and the data were analysed using the same software and algorithms as described in the Experimental Section. The data show very low probability for non-specific interaction – only less than 7% of all force-distance curves displayed rupture events (Figure S2b). It is indicative that only very few of these rupture events displayed the characteristic stretching of the PEG-linker used to attached the to the AFM probe (most of the non-specific interactions are between the actual AFM probe and the substrate). The vast majority of the force-distance curves did not display any tip-sample interaction upon the retraction from the sample surface. The most probable reason for the very low non-specific interaction in our experiment is the fact that during the substrate functionalisation the SiOx surface is actually passivated (see Experimental Section) with the only option for attachment to the surface through His-Ni^{2+}-NTA coordination bonds. This strategy minimises the non-specific interaction of probe-attached RC-His_{12}-LH1-PufX and the substrate.

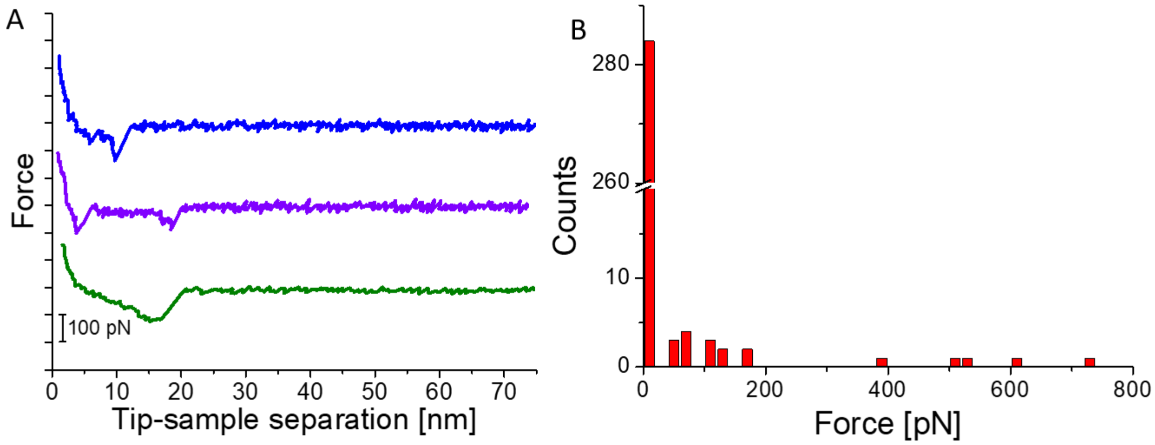

Figure S2. Negative control to show the lack of non-specific interaction between a functionalised, but cytochrome-free, substrate and the RC-attached probe

(**A**) Typical force-distance curves recorded upon the retraction of the RC-His$_{12}$-LH1-PufX-functionalised probe from the blank (no cytochrome c_2-His$_6$ attached) Ni^{2+}-NTA Si substrate at retraction velocity of 500 nm s^{-1}. All three curves display rupture events at various rupture lengths and the rupture forces are in the range 70 – 120 pN (for clarity the curves are offset along the Y-axis, the scale bar for Y-axis is 100 pN). (**B**) – Force distribution (most probable rupture force) for the non-specific unbinding between the probe and the surface. The histogram indicates a binding probability of less than 7%.

References

1. Kienberger, F. *et al.* (2000) Static and dynamical properties of single poly(ethylene glycol) molecules investigated by force spectroscopy. *Single Mol.* **1**, 123-128

2. Marko, J. F. & Siggia, E. D. (1995) Stretching DNA. *Macromol.* **28**, 8759–8770.
